# Supplementary material for: Factors Associated with Supportive Care Needs Among Palestinian Women with Breast Cancer in the West Bank: A Cross-Sectional Study
Source: Cancers (Basel). 2024 Oct 30;16(21):3663. doi: 10.3390/cancers16213663 (PMC11545701; doi:10.3390/cancers16213663)
Supplement: Supplementary file 1 [file cancers-16-03663-s001.zip › cancers-3258086-supplementary.pdf]

Table S1. Supportive care (physical and daily living, psychological, and sexual) needs and socio-demographic variables.

Variables		Physical and daily living needs					Psychological needs					Sexual needs				
		No need		Unmet needs		Chi-square	No need		Unmet needs		Chi-square	No need		Unmet needs		Chi-square
		N	%	N	%	p-value	N	%	N	%	p-value	N	%	N	%	p-value
**Governorate**	Hebron	24	64.9%	220	67.7%	**0.73**	60	66.7%	184	67.6%	**0.86**	144	69.2%	100	64.9%	**0.38**
	Bethlehem	13	35.1%	105	32.3%		30	33.3%	88	32.4%		64	30.8%	54	35.1%	
**Residency**	Refugee Camps	5	13.5%	22	6.8%		11	12.2%	16	5.9%		18	8.7%	9	5.8%	
	Village	20	54.1%	195	60.0%	**0.33**	53	58.9%	162	59.6%	**0.12**	124	59.6%	91	59.1%	**0.54**
	City	12	32.4%	108	33.2%		26	28.9%	94	34.6%		66	31.7%	54	35.1%	
**Age (years)**	≤40	13	35.1%	65	20.0%		6	6.7%	72	26.5%		17	8.2%	61	39.6%	
	41-54	20	54.1%	166	51.1%	**0.02**	33	36.7%	153	56.3%	**<0.001**	107	51.4%	79	51.3%	**<0.001**
	≥55	4	10.8%	94	28.9%		51	56.7%	47	17.3%		84	40.4%	14	9.1%	
**Marital status**	Single	6	16.2%	22	6.8%		7	7.8%	21	7.7%		21	10.1%	7	4.5%	
	Married	30	81.1%	275	84.6%	**0.07**	73	81.1%	232	85.3%	**0.45**	163	78.4%	142	92.2%	**0.001**
	Divorced/widow	1	2.7%	28	8.6%		10	11.1%	19	7.0%		24	11.5%	5	3.2%	
**Education**	Primary and less	4	10.8%	58	17.8%		29	32.2%	33	12.1%		47	22.6%	15	9.7%	
	Secondary	19	51.4%	144	44.3%		33	36.7%	130	47.8%		96	46.2%	67	43.5%	
	Diploma	3	8.1%	28	8.6%	**0.72**	8	8.9%	23	8.5%	**<0.001**	20	9.6%	11	7.1%	**<0.001**
	University and above	11	29.7%	95	29.2%		20	22.2%	86	31.6%		45	21.6%	61	39.6%	
**Working status**	Employee	11	29.7%	62	19.1%		12	13.3%	61	22.4%		35	16.8%	38	24.7%	
	Housewife	26	70.3%	254	78.2%	**0.21**	72	80.0%	208	76.5%	**0 .004**	164	78.8%	116	75.3%	**0.01**
	Retired	0	0.0%	9	2.8%		6	6.7%	3	1.1%		9	4.3%	0	0.0%	
**Number of children**	≤5	27	73.0%	218	67.1%	**0.47**	60	66.7%	185	68.0%	**0.81**	133	63.9%	112	72.7%	**0.08**
	>5	10	27.0%	107	32.9%		30	33.3%	87	32.0%		75	36.1%	42	27.3%	
**Family size**	≤5	19	51.4%	113	34.8%	**0.04**	31	34.4%	101	37.1%	**0.65**	67	32.2%	65	42.2%	**0.05**
	>5	18	48.6%	212	65.2%		59	65.6%	171	62.9%		141	67.8%	89	57.8%	
**Monthly Income (US $)**	<570	12	32.4%	151	46.5%		45	50.0%	118	43.4%		106	51.0%	57	37.0%	
	570-1140	16	43.2%	122	37.5%	**0.21**	34	37.8%	104	38.2%	**0.34**	72	34.6%	66	42.9%	**0.03**
	>1140	9	24.3%	52	16.0%		11	12.2%	50	18.4%		30	14.4%	31	20.1%	

Table S2. Supportive care (patient care and support, health information, and total) needs and socio-demographic variables.

**Variables**		**Patient care and support needs**					**Health information needs**					**Total**				
		**No need**		**Unmet needs**		**Chi-square**	**No need**		**Unmet needs**		**Chi-square**	**No need**		**Unmet needs**		**Chi-square**
		**N**	**%**	**N**	**%**	**p-value**	**N**	**%**	**N**	**%**	**p-value**	**N**	**%**	**N**	**%**	**p-value**
**Governorate**	Hebron	40	60.6%	204	68.9%	**0. 19**	16	69.6%	228	67.3%	**0.82**	94	66.7%	150	67.9%	**0.81**
	Bethlehem	26	39.4%	92	31.1%		7	30.4%	111	32.7%		47	33.3%	71	32.1%	
**Residency**	Refugee Camps	5	7.6%	22	7.4%	**0.03**	2	8.7%	25	7.4%	**0.25**	12	8.5%	15	6.8%	**0.29**
	Village	48	72.7%	167	56.4%		17	73.9%	198	58.4%		89	63.1%	126	57.0%	
	City	13	19.7%	107	36.1%		4	17.4%	116	34.2%		40	28.4%	80	36.2%	
**Age (years)**	≤40	7	10.6%	71	24.0%	**<0.001**	2	8.7%	76	22.4%	**0.01**	14	9.9%	64	29.0%	**<0.001**
	41-54	27	40.9%	159	53.7%		9	39.1%	177	52.2%		72	51.1%	114	51.6%	
	≥55	32	48.5%	66	22.3%		12	52.2%	86	25.4%		55	39.0%	43	19.5%	
**Marital status**	Single	7	10.6%	21	7.1%	**0.22**	4	17.4%	24	7.1%	**0.11**	14	9.9%	14	6.3%	**0.46**
	Married	51	77.3%	254	85.8%		16	69.6%	289	85.3%		116	82.3%	189	85.5%	
	Divorced/widow	8	12.1%	21	7.1%		3	13.0%	26	7.7%		11	7.8%	18	8.1%	
**Education**	Primary and less	19	28.8%	43	14.5%	**0.004**	9	39.1%	53	15.6%	0.03	32	22.7%	30	13.6%	**0.04**
	Secondary	27	40.9%	136	45.9%		8	34.8%	155	45.7%		66	46.8%	97	43.9%	
	Diploma	9	13.6%	22	7.4%		1	4.3%	30	8.8%		11	7.8%	20	9.0%	
	University and above	11	16.7%	95	32.1%		5	21.7%	101	29.8%		32	22.7%	74	33.5%	
**Working status**	Employee	11	16.7%	62	20.9%	**0.61**	4	17.4%	69	20.4%	**0.67**	27	19.1%	46	20.8%	**0.56**
	Housewife	54	81.8%	226	76.4%		19	82.6%	261	77.0%		109	77.3%	171	77.4%	
	Retired	1	1.5%	8	2.7%		0	0.0%	9	2.7%		5	3.5%	4	1.8%	
**Number of children**	≤5	48	72.7%	197	66.6%	**0.33**	16	69.6%	229	67.6%	**0.84**	94	66.7%	151	68.3%	**0.74**
	>5	18	27.3%	99	33.4%		7	30.4%	110	32.4%		47	33.3%	70	31.7%	
**Family size**	≤5	25	37.9%	107	36.1%	**0.79**	6	26.1%	126	37.2%	**0.28**	47	33.3%	85	38.5%	**0.32**
	>5	41	62.1%	189	63.9%		17	73.9%	213	62.8%		94	66.7%	136	61.5%	
**Monthly Income (US $)**	<570	40	60.6%	123	41.6%	**0.01**	14	60.9%	149	44.0%	**0.16**	67	47.5%	96	43.4%	**0.65**
	570-1140	20	30.3%	118	39.9%		8	34.8%	130	38.3%		53	37.6%	85	38.5%	
	>1140	6	9.1%	55	18.6%		1	4.3%	60	17.7%		21	14.9%	40	18.1%	

Table S3. Clinical characteristics, social support, and supportive care (physical and daily living, psychological, and sexual) needs.

Variables		Physical and daily living needs					Psychological needs					Sexuality needs				
		No need		Unmet needs		Chi-square	No need		Unmet needs		Chi-square	No need		Unmet needs		Chi-square
		N	%	N	%	p-value	N	%	N	%	p-value	N	%	N	%	p-value
**Duration of having breast cancer**	< 6 months	10	27.0%	80	24.6%	**0.125**	10	11.1%	80	29.4%	**<0.001**	37	17.8%	53	34.4%	**<0.001**
	6 months- 1 year	1	2.7%	48	14.8%		9	10.0%	40	14.7%		23	11.1%	26	16.9%	
	> 1 year	26	70.3%	197	60.6%		71	78.9%	152	55.9%		148	71.2%	75	48.7%	
**Stage of breast cancer at time of diagnosis**	Stage 1	3	8.1%	21	6.5%	**0.43**	10	11.1%	14	5.1%	**0.16**	12	5.8%	12	7.8%	**0.157**
	Stage 2	16	43.2%	107	32.9%		27	30.0%	96	35.3%		62	29.8%	61	39.6%	
	Stage 3	16	43.2%	156	48.0%		40	44.4%	132	48.5%		108	51.9%	64	41.6%	
	Stage 4	2	5.4%	41	12.6%		13	14.4%	30	11.0%		26	12.5%	17	11.0%	
**Family relative history of cancer**	Breast cancer	10	27.0%	90	27.7%	**0.97**	24	26.7%	76	27.9%	**0.95**	48	23.1%	52	33.8%	**0.07**
	Other cancers	8	21.6%	74	22.8%		20	22.2%	62	22.8%		51	24.5%	31	20.1%	
	No cancer history	19	51.4%	161	49.5%		46	51.1%	134	49.3%		109	52.4%	71	46.1%	
**Do you have chronic diseases?**	No	30	81.1%	206	63.4%	**0.03**	45	50.0%	191	70.2%	**<0.001**	122	58.7%	114	74.0%	**0. 002**
	Yes	7	18.9%	119	36.6%		45	50.0%	81	29.8%		86	41.3%	40	26.0%	
**Do you take pain medication?**	No	29	78.4%	151	46.5%	**<0.001**	44	48.9%	136	50.0%	**0.85**	103	49.5%	77	50.0%	**0.93**
	Yes	8	21.6%	174	53.5%		46	51.1%	136	50.0%		105	50.5%	77	50.0%	
**Chemotherapy**	No	4	10.8%	11	3.4%	**0.03**	4	4.4%	11	4.0%	**0.87**	7	3.4%	8	5.2%	**0.39**
	Yes	33	89.2%	314	96.6%		86	95.6%	261	96.0%		201	96.6%	146	94.8%	
**Radiation therapy**	No	12	32.4%	104	32.0%	**0.96**	20	22.2%	96	35.3%	**0.02**	52	25.0%	64	41.6%	**0.001**
	Yes	25	67.6%	221	68.0%		70	77.8%	176	64.7%		156	75.0%	90	58.4%	
**Hormone therapy**	No	16	43.2%	152	46.8%	**0.68**	28	31.1%	140	51.5%	**0.001**	83	39.9%	85	55.2%	**0.004**
	Yes	21	56.8%	173	53.2%		62	68.9%	132	48.5%		125	60.1%	69	44.8%	
**Biological therapy**	No	27	73.0%	197	60.6%	**0.14**	50	55.6%	174	64.0%	**0.15**	113	54.3%	111	72.1%	**0.001**
	Yes	10	27.0%	128	39.4%		40	44.4%	98	36.0%		95	45.7%	43	27.9%	
**Surgical therapy**	No	11	29.7%	56	17.2%	**0.06**	12	13.3%	55	20.2%	**0.14**	31	14.9%	36	23.4%	**0.04**
	Yes	26	70.3%	269	82.8%		78	86.7%	217	79.8%		177	85.1%	118	76.6%	
**Surgical intervention**	Complete mastectomy	17	45.9%	102	31.4%	**0.19**	28	31.1%	91	33.5%	**0.58**	64	30.8%	55	35.7%	**0.12**
	Partial mastectomy	15	40.5%	162	49.8%		48	53.3%	129	47.4%		111	53.4%	66	42.9%	
	No surgical intervention	5	13.5%	61	18.8%		14	15.6%	52	19.1%		33	15.9%	33	21.4%	
**Family and sons support**	No	2	5.4%	54	16.6%	**0.07**	12	13.3%	44	16.2%	**0.51**	30	14.4%	26	16.9%	**0.52**
	Yes	35	94.6%	271	83.4%		78	86.7%	228	83.8%		178	85.6%	128	83.1%	
**Husband or partner support**	No	25	67.6%	244	75.1%	**0.32**	68	75.6%	201	73.9%	**0.75**	171	82.2%	98	63.6%	**<0.001**
	Yes	12	32.4%	81	24.9%		22	24.4%	71	26.1%		37	17.8%	56	36.4%	
**Medical staff support**	No	37	100.0%	319	98.2%	**0.40**	89	98.9%	267	98.2%	**0.64**	206	99.0%	150	97.4%	**0.22**
	Yes	0	0.0%	6	1.8%		1	1.1%	5	1.8%		2	1.0%	4	2.6%	
**Other sources of support**	No	32	86.5%	247	76.0%	**0.15**	69	76.7%	210	77.2%	**0.91**	159	76.4%	120	77.9%	**0.74**
	Yes	5	13.5%	78	24.0%		21	23.3%	62	22.8%		49	23.6%	34	22.1%	

Table S4. Clinical characteristics, social support and supportive care (patient care and support, health information, and total) needs.

**Variables**		**Patient care and support needs**					**Health information needs**					**Total**				
		**No need**		**Unmet needs**		**Chi-square**	**No need**		**Unmet needs**		**Chi-square**	**No need**		**Unmet needs**		**Chi-square**
		**N**	**%**	**N**	**%**	**p-value**	**N**	**%**	**N**	**%**	**p-value**	**N**	**%**	**N**	**%**	**p-value**
**Duration of having breast cancer**	< 6 months	10	15.2%	80	27.0%	**0.09**	4	17.4%	86	25.4%	0.45	18	12.8%	72	32.6%	**<0.001**
	6 months- 1 year	8	12.1%	41	13.9%		2	8.7%	47	13.9%		19	13.5%	30	13.6%	
	> 1 year	48	72.7%	175	59.1%		17	73.9%	206	60.8%		104	73.8%	119	53.8%	
**Stage of breast cancer at time of diagnosis**	Stage 1	4	6.1%	20	6.8%	**0.78**	1	4.3%	23	6.8%	0.82	11	7.8%	13	5.9%	**0.91**
	Stage 2	26	39.4%	97	32.8%		7	30.4%	116	34.2%		47	33.3%	76	34.4%	
	Stage 3	29	43.9%	143	48.3%		13	56.5%	159	46.9%		66	46.8%	106	48.0%	
	Stage 4	7	10.6%	36	12.2%		2	8.7%	41	12.1%		17	12.1%	26	11.8%	
**Family relative history of cancer**	Breast cancer	13	19.7%	87	29.4%	**0.19**	3	13.0%	97	28.6%	0.21	32	22.7%	68	30.8%	**0.23**
	Other cancers	**14**	**21.2%**	**68**	**23.0%**		5	21.7%	77	22.7%		33	23.4%	49	22.2%	
	No cancer history	39	59.1%	141	47.6%		15	65.2%	165	48.7%		76	53.9%	104	47.1%	
**Do you have chronic diseases?**	No	36	54.5%	200	67.6%	**0.04**	13	56.5%	223	65.8%	0.36	83	58.9%	153	69.2%	**0.04**
	Yes	30	45.5%	96	32.4%		10	43.5%	116	34.2%		58	41.1%	68	30.8%	
**Do you take pain medication?**	No	35	53.0%	145	49.0%	**0.55**	12	52.2%	168	49.6%	0.80	79	56.0%	101	45.7%	**0.05**
	Yes	31	47.0%	151	51.0%		11	47.8%	171	50.4%		62	44.0%	120	54.3%	
**Chemotherapy**	No	2	3.0%	13	4.4%	**0.62**	1	4.3%	14	4.1%	0.09	6	4.3%	9	4.1%	**0.93**
	Yes	**64**	**97.0%**	**283**	**95.6%**		22	95.7%	325	95.9%		135	95.7%	212	95.9%	
**Radiation therapy**	No	15	22.7%	101	34.1%	**0.07**	7	30.4%	109	32.2%	**0.86**	34	24.1%	82	37.1%	**0.01**
	Yes	51	77.3%	195	65.9%		16	69.6%	230	67.8%		107	75.9%	139	62.9%	
**Hormone therapy**	No	21	31.8%	147	49.7%	**0.009**	7	30.4%	161	47.5%	**0.11**	48	34.0%	120	54.3%	**<0.001**
	Yes	45	68.2%	149	50.3%		16	69.6%	178	52.5%		93	66.0%	101	45.7%	
**Biological therapy**	No	35	53.0%	189	63.9%	**0.10**	15	65.2%	209	61.7%	**0.73**	75	53.2%	149	67.4%	**0.007**
	Yes	31	47.0%	107	36.1%		8	34.8%	130	38.3%		66	46.8%	72	32.6%	
**Surgical therapy**	No	8	12.1%	59	19.9%	**0.14**	4	17.4%	63	18.6%	**0.88**	20	14.2%	47	21.3%	**0.09**
	Yes	58	87.9%	237	80.1%		19	82.6%	276	81.4%		121	85.8%	174	78.7%	
**Surgical intervention**	Complete mastectomy	17	25.8%	102	34.5%	**0.24**	9	39.1%	110	32.4%	**0.63**	44	31.2%	75	33.9%	**0.48**
	Partial mastectomy	33	50.0%	144	48.6%		9	39.1%	168	49.6%		67	47.5%	110	49.8%	
	No surgical intervention	16	24.2%	50	16.9%		5	21.7%	61	18.0%		30	21.3%	36	16.3%	
**Family and sons support**	No	9	13.6%	47	15.9%	**0.65**	1	4.3%	55	16.2%	**0.13**	17	12.1%	39	17.6%	**0.15**
	Yes	57	86.4%	249	84.1%		22	95.7%	284	83.8%		124	87.9%	182	82.4%	
**Husband or partner support**	No	51	77.3%	218	73.6%	**0.54**	18	78.3%	251	74.0%	**0.65**	107	75.9%	162	73.3%	**0.58**
	Yes	15	22.7%	78	26.4%		5	21.7%	88	26.0%		34	24.1%	59	26.7%	
**Medical staff support**	No	64	97.0%	292	98.6%	**0.33**	22	95.7%	334	98.5%	**0.29**	137	97.2%	219	99.1%	**0.16**
	Yes	2	3.0%	4	1.4%		1	4.3%	5	1.5%		4	2.8%	2	0.9%	
**Other sources of support**	No	51	77.3%	228	77.0%	**0.96**	18	78.3%	261	77.0%	**0.88**	109	77.3%	170	76.9%	**0.93**
	Yes	15	22.7%	68	23.0%		5	21.7%	78	23.0%		32	22.7%	51	23.1%	
